# Epstein-Barr Virus Infection in Chronically Inflamed Periapical Granulomas

**DOI:** 10.1371/journal.pone.0121548

**Published:** 2015-04-17

**Authors:** Kosuke Makino, Osamu Takeichi, Keisuke Hatori, Kenichi Imai, Kuniyasu Ochiai, Bunnai Ogiso

**Affiliations:** 1 Department of Endodontics, Nihon University School of Dentistry, Chiyoda-ku, Tokyo, Japan; 2 Department of Microbiology, Nihon University School of Dentistry, Chiyoda-ku, Tokyo, Japan; The University of North Carolina at Chapel Hill, UNITED STATES

## Abstract

Periapical granulomas are lesions around the apex of a tooth caused by a polymicrobial infection. Treatment with antibacterial agents is normally performed to eliminate bacteria from root canals; however, loss of the supporting alveolar bone is typically observed, and tooth extraction is often selected if root canal treatment does not work well. Therefore, bacteria and other microorganisms could be involved in this disease. To understand the pathogenesis of periapical granulomas more precisely, we focused on the association with Epstein-Barr virus (EBV) using surgically removed periapical granulomas (n = 32). EBV DNA was detected in 25 of 32 periapical granulomas (78.1%) by real-time PCR, and the median number of EBV DNA copies was approximately 8,688.01/μg total DNA. In contrast, EBV DNA was not detected in healthy gingival tissues (n = 10); the difference was statistically significant according to the Mann-Whitney *U* test (*p* = 0.0001). Paraffin sections were also analyzed by *in situ* hybridization to detect EBV-encoded small RNA (EBER)-expressing cells. EBER was detected in the cytoplasm and nuclei of B cells and plasma cells in six of nine periapical granulomas, but not in healthy gingival tissues. In addition, immunohistochemical analysis for latent membrane protein 1 (LMP-1) of EBV using serial tissue sections showed that LMP-1-expressing cells were localized to the same areas as EBER-expressing cells. These data suggest that B cells and plasma cells in inflamed granulomas are a major source of EBV infection, and that EBV could play a pivotal role in controlling immune cell responses in periapical granulomas.

## Introduction

Periapical periodontitis is characterized by supporting tissue damage and progressive alveolar bone resorption around the apical areas of affected teeth [1]. It is caused by a mixed bacterial infection in the oral cavity, and dental caries is thought to be a major source of this infection [2]. Periodontopathic bacteria such as *Porphyromonus gingivalis*, *Fusobacterium nucleatum*, and *Eubacterium* species are microbiological research foci in dentistry because they produce lipopolysaccharide (LPS), a strong virulence factor [3].

Chronic inflammation due to periapical periodontitis may lead to the formation of periapical granulomas, which consist of granulomatous tissue containing inflammatory cells such as polymorphonuclear leukocytes (PMNs), lymphocytes, plasma cells, and macrophages. Large numbers of microvessels are also observed typically. Inflammatory cells, which express various cytokines and growth factors that augment immune defenses in response to LPS stimulation [4–6], appear to play an important role in the progression of periapical periodontitis. Interestingly, granulomatous tissue is surrounded by alveolar bone at the apex of teeth, and the source of infection is limited to the root canals of teeth. Thus, it is an ideal human model for assessing the relationship between infection and inflammation at sites of bone resorption.

A local drug delivery system using a combination of three antibiotics (minocycline, ciprofloxacin, and metronidazole) has been reported to eliminate periodontopathic bacteria through root canals and to be effective for the treatment of patients with periapical periodontitis [7]. Nevertheless, some patients are not healed following the application of antibiotics; such cases of chronic inflammation are referred to as persistent periapical periodontitis [8], suggesting that antibacterial drug-resistant microorganisms such as viruses or fungi could be present in the periapical lesions. Unfortunately, tooth extraction is often planned for the treatment of these patients. Thus, more effective pharmacological therapies must be developed to preserve teeth.

Epstein-Barr virus (EBV) is an oncogenic herpesvirus that infects a significant percentage (>90%) of the population worldwide and is the causative agent of infectious mononucleosis [9]. It is detected frequently in Burkitt’s lymphoma, Hodgkin’s disease, and T-cell lymphoma. Thus, EBV has been implicated in the pathogenesis of several malignancies. In contrast, a recent publication demonstrated an association between EBV and inflammatory reactions inducing cytokine expression by inflammatory cells [10]. A correlation between EBV infection and rheumatoid arthritis [11] and Sjogren syndrome [12] has also been shown. These observations suggest that EBV infection in periapical periodontitis could be related to tissue injury and cell damage; however, the existence of EBV infection in periapical lesions has not been examined. It is necessary to examine periapical lesions for EBV, because the virus could be important in periapical immune reactions. Confirmed EBV infection in periapical granulomas may also promote the development of novel pharmacological therapies. The purpose of this study was to assess whether EBV was present in periapical granulomas and inflammatory infiltrates are infected with EBV. To demonstrate the presence of an EBV infection, *in situ* hybridization (ISH) for EBV-encoded small RNA (EBER) was performed. EBER is expressed in latent EBV-infected cells and can be used as a target for the detection of EBV-infected cells in tissues [13]. We also examined the prevalence of EBV infection in periapical granulomas using quantitative real-time PCR for EBV DNA and immunohistochemistry for latent membrane protein 1 (LMP-1) of EBV. Healthy gingival tissues were also examined as a control.

## Materials and Methods

### Patients

**Fig 1:**
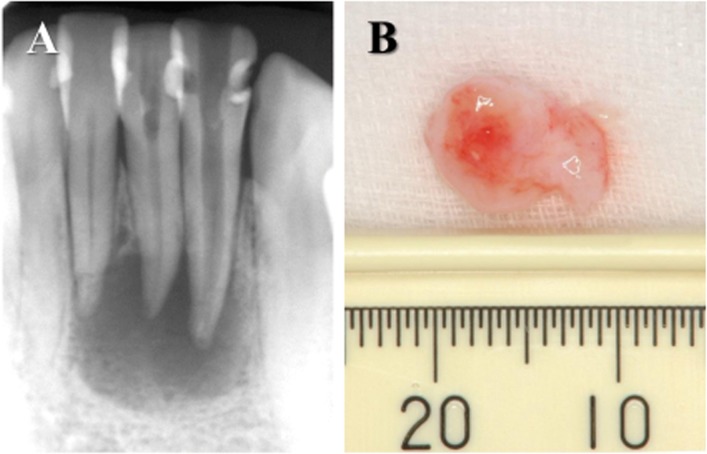
Specimens used in this study. (A) X-ray observation of periapical lesions caused at lower incisal teeth. Radiolucency around the apex showed alveolar bone resorption. (B) Periapical lesion surgically removed from a patient showing at (A).

In total, 40 patients (9 males and 31 females; age range, 24–78 years) who were referred to the Department of Endodontics at Nihon University School of Dentistry Dental Hospital (Tokyo, Japan) because of persistent periapical periodontitis were included in this study. The clinical symptoms of the patients included an absence of throbbing pain, pain on palpation of the mucosa around the root apex, and percussion pain. An apparent radiolucency was seen around the root apex of all teeth (Fig 1A). Endodontic treatments had been applied several times by general practitioners; however, the patients were still experiencing symptoms. Therefore, endodontic surgery (apicoectomy and retrograde filling) was selected. Patients had no systemic diseases, and antibiotics have not been taken during the previous 6 months. This study was approved by the ethics committee of Nihon University School of Dentistry, based on the Declaration of Helsinki. Before sample collection, the experimental design, risks, and potential for discomfort were explained fully, and all patients signed consent forms.

Healthy gingival tissues were obtained at the time of extraction of impacted wisdom teeth from 10 patients, who were referred to the Department of Oral Surgery. These patients had no clinical symptoms, such as pain or swelling, and an X-ray examination did not indicate inflammation.

### Pathological Examination of the Samples

The periapical lesions (Fig 1B) and healthy gingival tissues, which were removed from the patients at the time of surgical treatment, measured approximately 0.82 and 0.44 cm in diameter, respectively. The tissues were fixed immediately in acetone and then embedded in paraffin. Serial tissue sections (7 μm in thickness) were prepared. A pathological examination of the specimens was performed after hematoxylin and eosin staining. Sectioned areas were selected randomly, and at least three different regions in each sample were examined. The sections were investigated using a light microscope (Olympus BH-2, Tokyo, Japan). Periapical granulomas and healthy gingival tissues, which were confirmed pathologically, were utilized for the following studies.

### Quantitative Analysis of the EBV DNA Copy Number

EBV in the periapical granuloma and healthy gingival tissue samples was detected quantitatively using real-time PCR. In brief, DNA was extracted from paraffin sections of periapical granulomas or healthy gingival tissues using a QIAamp DNA Mini Kit (Qiagen, Hilden, Germany). In total, 40 ng of each DNA sample was amplified in a 25-μl reaction mixture containing SYBR Premix Ex *Taq* (Takara Bio Inc., Otsu, Japan) and 20 μM each of the sense and antisense PCR primers for EBV. The sequences of the primers were TGCTTCGTTATAGCCGTAGT (reverse) and CCTGGTCATCCTTTGCCA (forward). Amplification was conducted in 40 cycles of 95°C for 15 s and 60°C for 60 s using a Smart Cycler (Cepheid, Sunnyvale, CA, USA).

For the preparation of a standard curve to determine the EBV DNA copy number, a 10-fold serial dilution of a known quantity of EBV DNA (approximately 1x10^6^ copies/μl) was prepared. Each diluted DNA sample was amplified using real-time PCR alongside the DNA extracted from the specimens. A standard curve was prepared using semi-log graphs, and the EBV DNA copy number in the specimens was calculated.

### ISH to Detect EBER-Expressing Cells

ISH using EBER-specific probes was employed, and periapical granulomas (n = 9), found to be EBV-positive by real-time PCR and healthy gingival tissues (n = 5) were examined. In brief, deparaffinized sections were digested with proteinase K for 30 min and then hybridized with hapten 5-carboxy-fluorescein-labeled 15-nucleotide single-stranded DNA probes for EBV (Dako, Glostrup, Denmark) at 55° for 90 min. After a stringent wash with 0.5× SSC, alkaline phosphatase-conjugated anti-FITC rabbit polyclonal antibodies (Dako) were incubated with the mixture for 30 min. Finally, a colorimetric reaction using DAB was performed, followed by counterstaining with hematoxylin. Tissue sections of human malignant lymphomas were used as a positive control for EBER ISH. The negative control constituted hybridization without the addition of the EBER probe.

### Immunohistochemical Analysis

To examine the localization of LMP-1-expressing cells, periapical granulomas (n = 6) confirmed for EBER mRNA expression by ISH and healthy gingival tissues (n = 5) were investigated. Serial paraffin sections of EBER-positive specimens were analyzed. In brief, the paraffin sections were deparaffinized, rehydrated, and incubated with 0.3% H_2_O_2_ in methanol for 30 min to quench endogenous peroxidase. The sections were incubated first with 10% normal horse serum (Vector Laboratories, Burlingame, CA, USA) to block non-specific binding for 20 min, followed by human LMP-1 monoclonal antibodies (1:100 in PBS; Dako), followed by biotinylated anti-mouse IgG antibodies (Vector Laboratories), and finally with avidin-biotin peroxidase complex. To reveal LMP-1-positive cells via color development, the horseradish peroxidase substrate (3,3'-diaminobenzidine; Vector Laboratories) was used, and hematoxylin counterstaining was performed. Normal mouse IgG antibodies (Cayman Chemical, Ann Arbor, MI, USA) were used as a negative control in place of anti-LMP-1 antibodies.

### Statistical Analysis

EBV detection using real-time PCR was analyzed statistically using SPSS version 15.0 for Windows (SPSS, Chicago, IL). The comparison between periapical granulomas and healthy gingival tissues was made using Mann-Whitney *U*-test.

## Results

### Pathological Examination of the Samples

**Fig 2:**
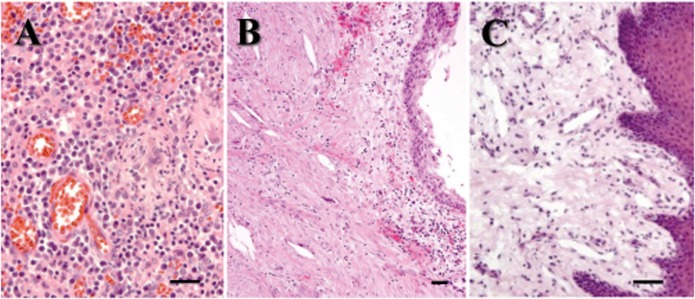
Histological evaluations of the specimens. Paraffin sections (n = 40) were stained using hematoxylin and eosin. Scale bar = 100μm. (A) Periapical granulomas (n = 32) showing a large number of inflammatory cells and microvessels. (B) Radicular cyst (n = 8) showing epithelial cell layer and cholesterol clefts. (C) Healthy gingival tissues (n = 10) showing epithelial cell layer and lower cell number of infiltrating cells in comparison with periapical granulomas.

Surgically removed periapical lesions were examined pathologically. Of 40 periapical lesions stained with hematoxylin and eosin, 32 exhibited granulomatous tissues with large numbers of microvessels and inflammatory cells such as PMNs, lymphocytes, plasma cells, and macrophages (Fig 2A). No epithelial cells were observed, and the specimens were diagnosed as periapical granulomas. The remaining periapical lesions exhibited an epithelial cell lining with cholesterol clefts in granulomatous tissue and were diagnosed as radicular cysts (Fig 2B); these were not used in this study. The healthy gingival tissues contained more collagen fibers and fewer inflammatory cells in the granulomatous tissue than did the periapical granulomas (Fig 2C).

### Quantitative Analysis of EBV DNA Copy Numbers

**Fig 3:**
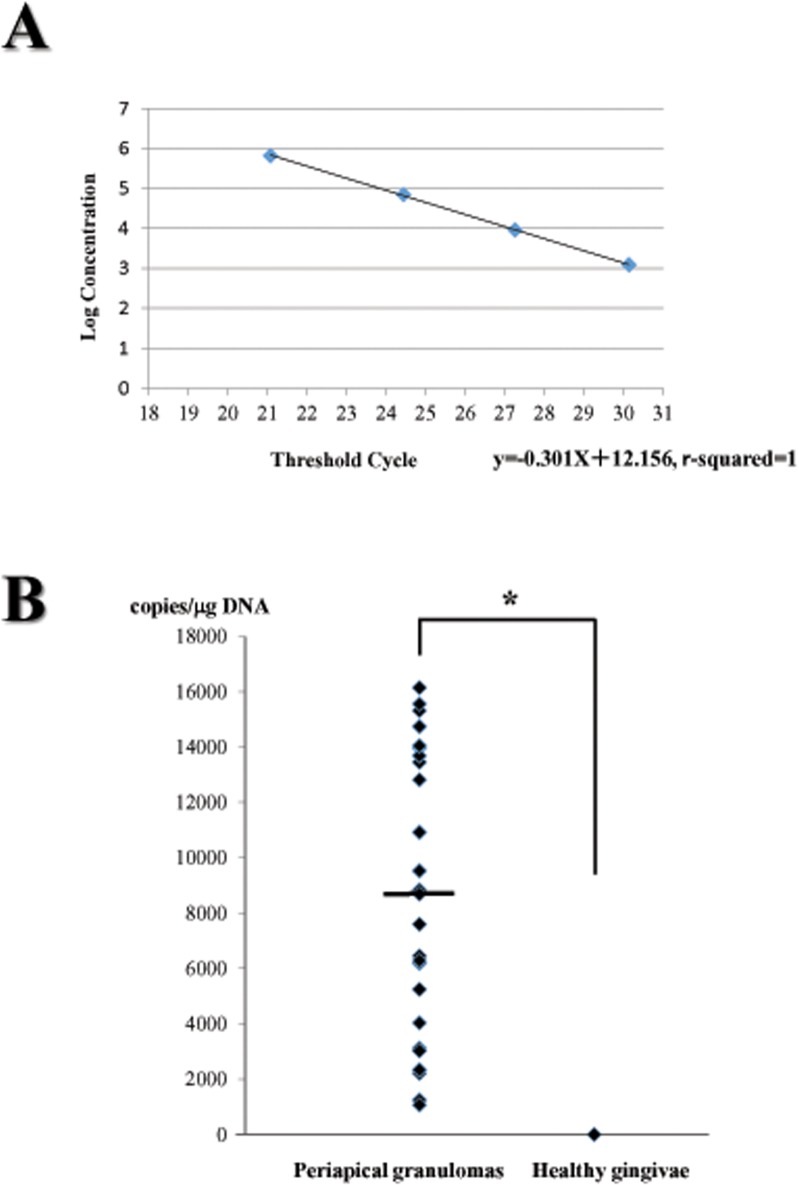
Quantitative real time PCR analysis. (A) Standard curve to determine the copies of EBV DNA in specimens. EBV DNA (approximately 1x10^6^ copies/μl) was diluted in ten-fold serially. Each diluted DNA was amplified using real-time PCR simultaneously at the time of amplification for DNA extracted from periapical granulomas and healthy gingivae. (B) Detection of EBV DNA in periapical granulomas and healthy gingivae. The copy of EBV DNA in each specimen was calculated using the standard curve. The median of EBV DNA copies in periapical granulomas was approximately 8688.01 per 1μg of total DNA, as shown by a horizontal bar. * showed statistical difference using Mann-Whitney *U* test (*p* = 0.0001).

DNA extracted from periapical granulomas and healthy gingival tissues was amplified using real-time PCR with EBV-specific primers. A standard curve was prepared after PCR amplification using serially diluted EBV DNA, as shown in Fig 3A, and PCR was performed at the same time as the amplification of DNA from periapical granulomas and healthy gingival tissues.

Of 32 periapical granulomas, 25 specimens (78.1%) exhibited the presence of EBV (Table 1). The median EBV DNA copy number was approximately 8,688.01/μg total DNA (Fig 3B). In contrast, EBV DNA was not detected in the healthy gingival tissues. A statistical analysis demonstrated that the EBV detection rate in periapical granulomas was significantly higher than that in healthy gingival tissues (*p* = 0.0001).

**Table 1:** Detection of EBV DNA using quantitative real-time PCR.

	periapical granulomas (n = 32)	healthy gingival tissues (n = 10)	P-value
**presence**	25 (78.1%)	0 (0.0%)	0.0001*
**absence**	7 (21.9%)	10 (100.0%)	

Periapical granulomas and healthy gingival tissues were analyzed to detect EBV DNA by quantitative real-time PCR. EBV DNA were highly detected from periapical granulomas in comparison with healthy gingival tissues.

* Mann-Whitney *U* test.

### ISH for EBER and Immunohistochemistry for LMP-1

**Fig 4:**
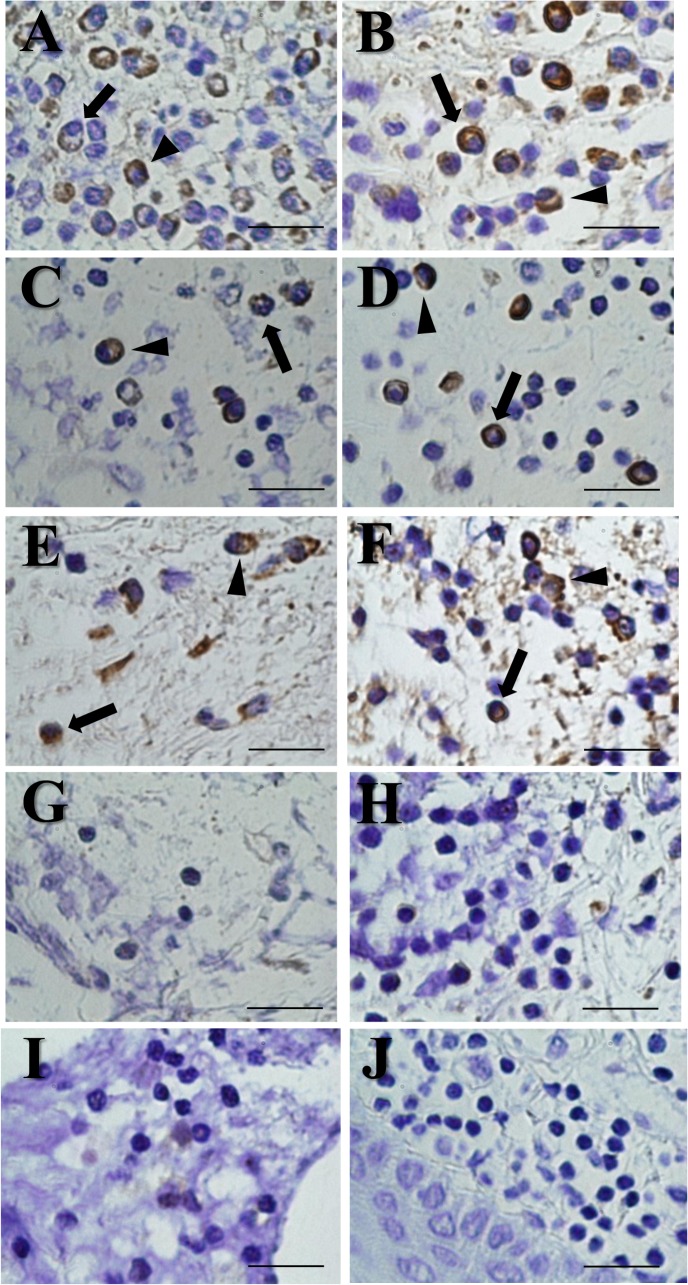
Detection of EBER using *in situ* hybridization. (A-F) *In situ* detection of EBER-expressing cells in periapical granulomas (6 out of 9) was shown in the cytoplasm and nuclei of B cells (arrows) and plasma cells (arrow heads). (G-I) Three periapical granulomas showed EBER-negative expression. (J) Healthy gingival tissues never showed positive expression. Scale bar = 50μm.

*In situ* detection using EBER-specific probes was performed for periapical granulomas and healthy gingival tissues. In total, six of nine periapical granulomas (66.7%) showed positive expression of EBER (Fig 4A–4F). EBER localization was exhibited in the nucleus and cytoplasm of B lymphocytes and plasma cells. In contrast, EBV-negative periapical granulomas (Fig 4G–4I) and healthy gingival tissues (Fig 4J) did not show EBER positivity. No expression was detected using a sense probe for EBER (data not shown).

**Fig 5:**
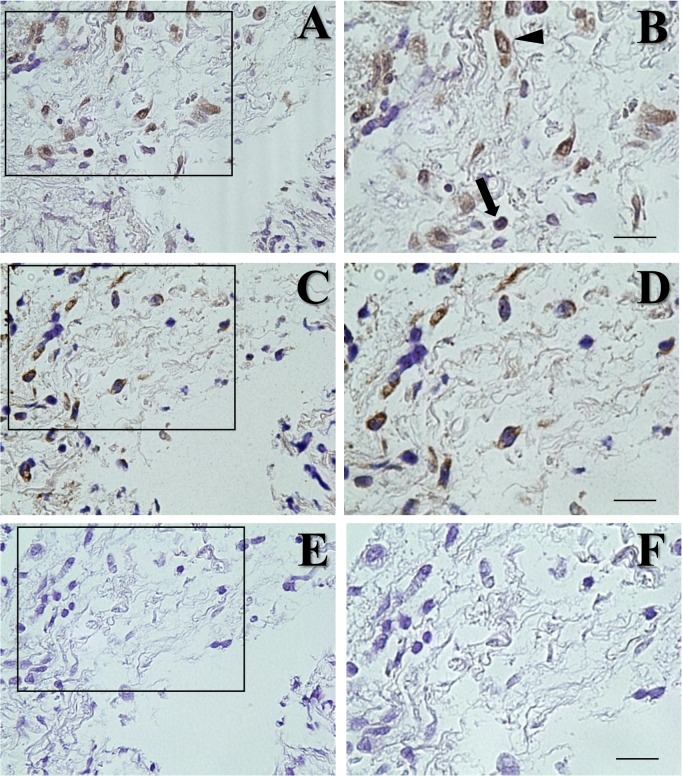
LMP-1 immunohistochemistry using serial sections of periapical granulomas. Framed boxes in left panels (A, C, E) were magnified to right panels (B, D, F, respectively). (A, B) LMP-1 immunohistochemistry showed positive staining in B cells (arrows) and plasma cells (arrow heads). (C, D) EBER-expressing cells were present at the same area of LMP-1-expressing cells. (E, F) A negative control using normal mouse IgG antibody did not exhibit LMP-1 expression. Scale bar = 50μm.

Immunohistochemistry for LMP-1 was also performed in periapical granulomas and healthy gingival tissues. All of the EBER-positive periapical granulomas that were confirmed by ISH showed positive staining for LMP-1 (Fig 5A and 5B). LMP-1 was expressed by B lymphocytes and plasma cells. In addition, EBER-expressing cells were localized to the same areas as LMP-1-expressing cells, based on serial tissue sections (Fig 5C and 5D). A negative control using normal mouse IgG antibody did not exhibit LMP-1 expression (Fig 5E and 5F). Healthy gingival tissues showed negative expression of LMP-1 (data not shown).

## Discussion

In this study, EBV was detected in 78.1% (25 of 32 samples) of periapical granulomas by real-time PCR. The presence of EBV in periapical lesions has been examined by immunohistochemistry for EBNA or PCR [14–16], and the EBV detection rate varied from 54.6–85.7%. Possible reasons for the variation among previous studies include differences in EBV detection methods, disease status, or regional features of EBV infection. Our EBV detection rates were relatively high compared with those in published data. We also determined the EBV DNA copy numbers in periapical granulomas, which have not yet been reported. The median EBV DNA copy number in periapical granulomas was 8,688.01/μg total DNA; in contrast, EBV was not detected in healthy tissues. Thus, periapical granulomas include extremely high levels of EBV DNA. EBV may be important in the initiation of periapical inflammation or may prolong apical inflammation as a result of increasing tissue damage.

EBV causes systemic diseases such as infectious mononucleosis. On the other hand, EBV induces immune dysregulation by inhibiting the replication of stimulated peripheral blood mononuclear leukocytes [17]. In addition, it up-regulates proinflammatory cytokines such as tumor necrosis factor-α, interleukin (IL)-1β, IL-6, IL-8, and IL-10 [18–20]. IL-10 is produced by T helper type-2 (Th2) cells, monocytes, macrophages, and B cells, and it inhibits the production of cytokines such as IL-2 and interferon-γ by Th1 cells [21]. Th-1 cytokines are important in controlling viral infections, and a shift in the cytokine profile toward Th2 could be favorable for virus-induced inflamed lesions. Thus, EBV infection may play a role in not only systemic but also local, inflammatory disease. In our study, EBV DNA was absent from healthy gingival tissues, suggesting that elevated levels of EBV in periapical granulomas contribute to immune reactions in periapical lesions, including tissue destruction and bone resorption. These data also indicate that local, inflamed lesions could be an EBV reservoir for systemic infections, supplying the virus through the blood via microvessels in granulomatous tissues.

Although the presence of EBV in periapical lesions has been examined [14–16], it has not been confirmed. In this study, EBER was detected in B cells and plasma cells in periapical granulomas using ISH, whereas healthy gingival tissues did not show EBER expression. Our data suggest that in periapical granulomas, these cells were infected with EBV because the presence of EBER is considered a reliable marker for the existence of latent EBV in cells [13]. We found that EBER was expressed in the nucleus and cytoplasm of cells. EBER expression has been detected in the nuclei of B cells in gastric carcinoma or tonsillar tissues from patients with tonsillitis by ISH [22,23]. However, Schwemmle *et al*. [24] demonstrated that EBER was located in both the cytoplasm and nucleus of cells using ISH and confocal laser microscopy. EBER was found to be secreted in a complex with La protein [25]. Thus, EBER forms complexes with cytoplasmic proteins and could localize to both the nucleus and the cytoplasm.

Immunohistochemistry using serial tissue sections demonstrated the presence of LMP-1-expressing cells in the same regions as EBER-expressing cells, in accordance with our EBER ISH results. On the other hand, the EBER expression rate in periapical granulomas was 66.7% for EBV-positive specimens confirmed by real-time PCR. The reason for the lower detection rate by ISH could be that the sensitivity of ISH is lower than that of real-time PCR.

The functional role of EBER in periapical granulomas is unclear; however, it has been demonstrated that EBER induces transcription of cytokines, including IL-10, insulin-like growth factor-1, and IL-9 [26–28]. It also contributes to the pathogenesis of EBV infection through modulation of innate immune signals [29]. Therefore, EBER-induced activation of innate immunity could account for immunopathologic diseases caused by EBV infection and could influence the pathogenesis of EBV-induced diseases. In addition, EBV-associated IL-10 expression has been detected in periapical lesions [30]. Thus, EBER could be associated with the pathogenesis of periapical granulomas by way of subsequent immune activation. Taken together, we conclude that B cells and plasma cells in periapical granulomas are major targets for EBV infection, and that these cells may play roles in various aspects of periapical inflammation. In addition, periapical granulomas could act as a reservoir for EBV to provide it systemically.

Recent studies have demonstrated that antimicrobial therapy for periodontal disease can reduce the presence of herpesviruses in periodontal tissues [31,32]. The reason might be associated with bacterial butyrate, histone deacetylase inhibitors, which can promote lytic reactivation from latency of EBV [33]. On the basis of published evidence and our data, we hypothesize that inhibition of viral replication or EBER secretion could be a novel therapeutic target for treatment of periapical periodontitis. Animal studies should be performed to confirm this hypothesis.

## Supporting Information

S1 Fig Specimens used in this study. (A) X-ray observation of periapical lesions caused at lower incisal teeth. Radiolucency around the apex showed alveolar bone resorption. (B) Periapical lesion surgically removed from a patient showing at (A).
(TIF)

S2 Fig Histological evaluations of the specimens. Paraffin sections (n = 40) were stained using hematoxylin and eosin. Scale bar = 100μm. (A) Periapical granulomas (n = 32) showing a large number of inflammatory cells and microvessels. (B) Radicular cyst (n = 8) showing epithelial cell layer and cholesterol clefts. (C) Healthy gingival tissues (n = 10) showing epithelial cell layer and lower cell number of infiltrating cells in comparison with periapical granulomas.
(TIF)

S3 Fig Quantitative real time PCR analysis. (A) Standard curve to determine the copies of EBV DNA in specimens. EBV DNA (approximately 1x10^6^ copies/μl) was diluted in ten-fold serially. Each diluted DNA was amplified using real-time PCR simultaneously at the time of amplification for DNA extracted from periapical granulomas and healthy gingivae. (B) Detection of EBV DNA in periapical granulomas and healthy gingivae. The copy of EBV DNA in each specimen was calculated using the standard curve. The median of EBV DNA copies in periapical granulomas was approximately 8688.01 per 1μg of total DNA, as shown by a horizontal bar. * showed statistical difference using Mann-Whitney *U* test (*p* = 0.0001).
(TIF)

S4 Fig Detection of EBER using *in situ* hybridization. (A-F) *In situ* detection of EBER-expressing cells in periapical granulomas (6 out of 9) was shown in the cytoplasm and nuclei of B cells (arrows) and plasma cells (arrow heads). (G-I) Three periapical granulomas showed EBER-negative expression. (J) Healthy gingival tissues never showed positive expression. Scale bar = 50μm.
(TIF)

S5 Fig LMP-1 immunohistochemistry using serial sections of periapical granulomas. Framed boxes in left panels (A, C, E) were magnified to right panels (B, D, F, respectively). (A, B) LMP-1 immunohistochemistry showed positive staining in B cells (arrows) and plasma cells (arrow heads). (C, D) EBER-expressing cells were present at the same area of LMP-1-expressing cells. (E, F) A negative control using normal mouse IgG antibody did not exhibit LMP-1 expression. Scale bar = 50μm.
(TIF)

S1 Table Detection of EBV DNA using quantitative real-time PCR. Periapical granulomas and healthy gingival tissues were analyzed to detect EBV DNA by quantitative real-time PCR. EBV DNA were highly detected from periapical granulomas in comparison with healthy gingival tissues. * Mann-Whitney *U* test.
(TIF)
