# Supplementary figures and images for: Epstein-Barr Virus Infection in Chronically Inflamed Periapical Granulomas
Source: PLoS One. 2015 Apr 17;10(4):e0121548. doi: 10.1371/journal.pone.0121548 (PMC4401687; doi:10.1371/journal.pone.0121548)

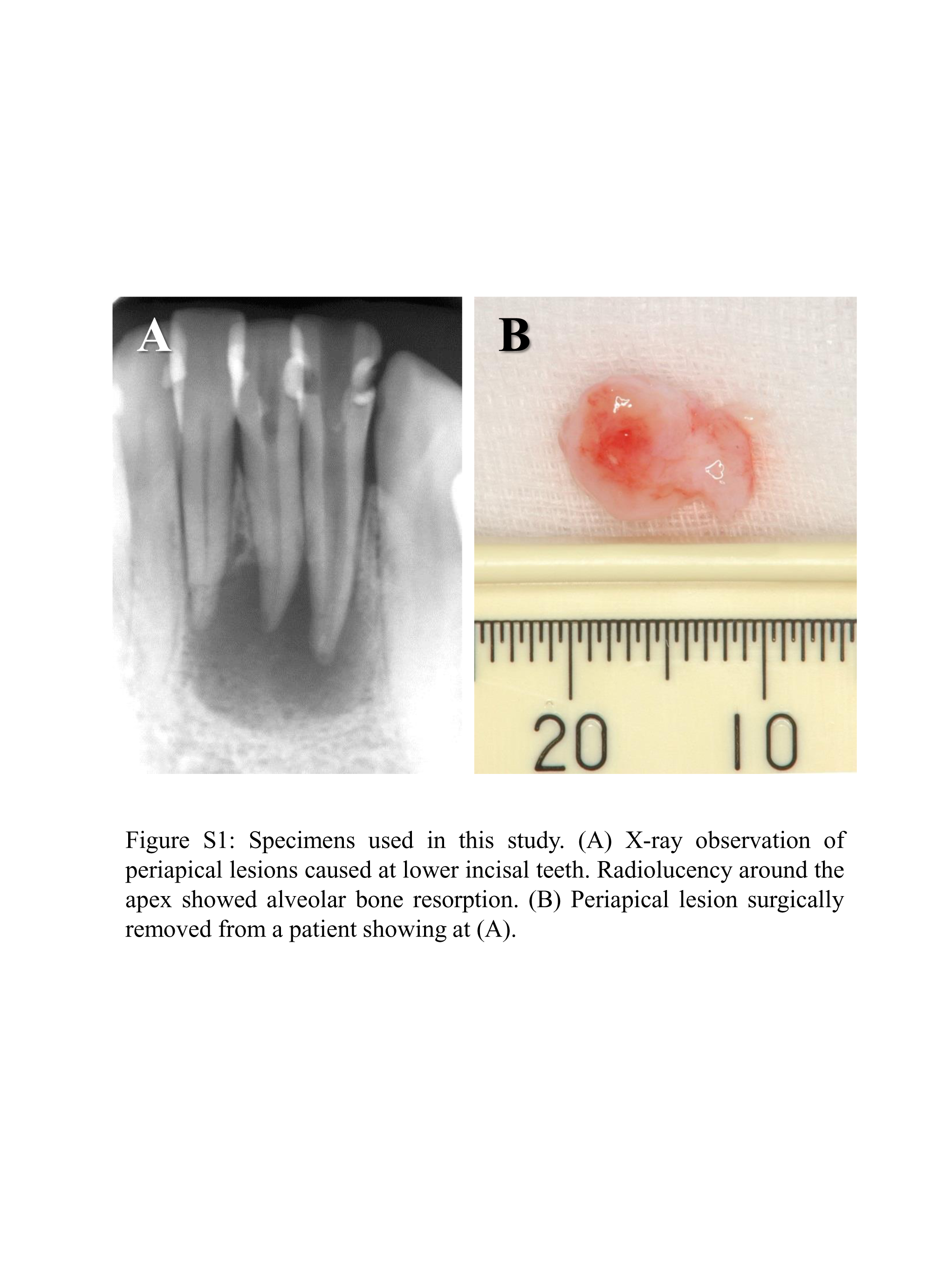

Supplement: S1 Fig — (A) X-ray observation of periapical lesions caused at lower incisal teeth. Radiolucency around the apex showed alveolar bone resorption. (B) Periapical lesion surgically removed from a patient showing at (A). (TIF) [file pone.0121548.s001.tif]

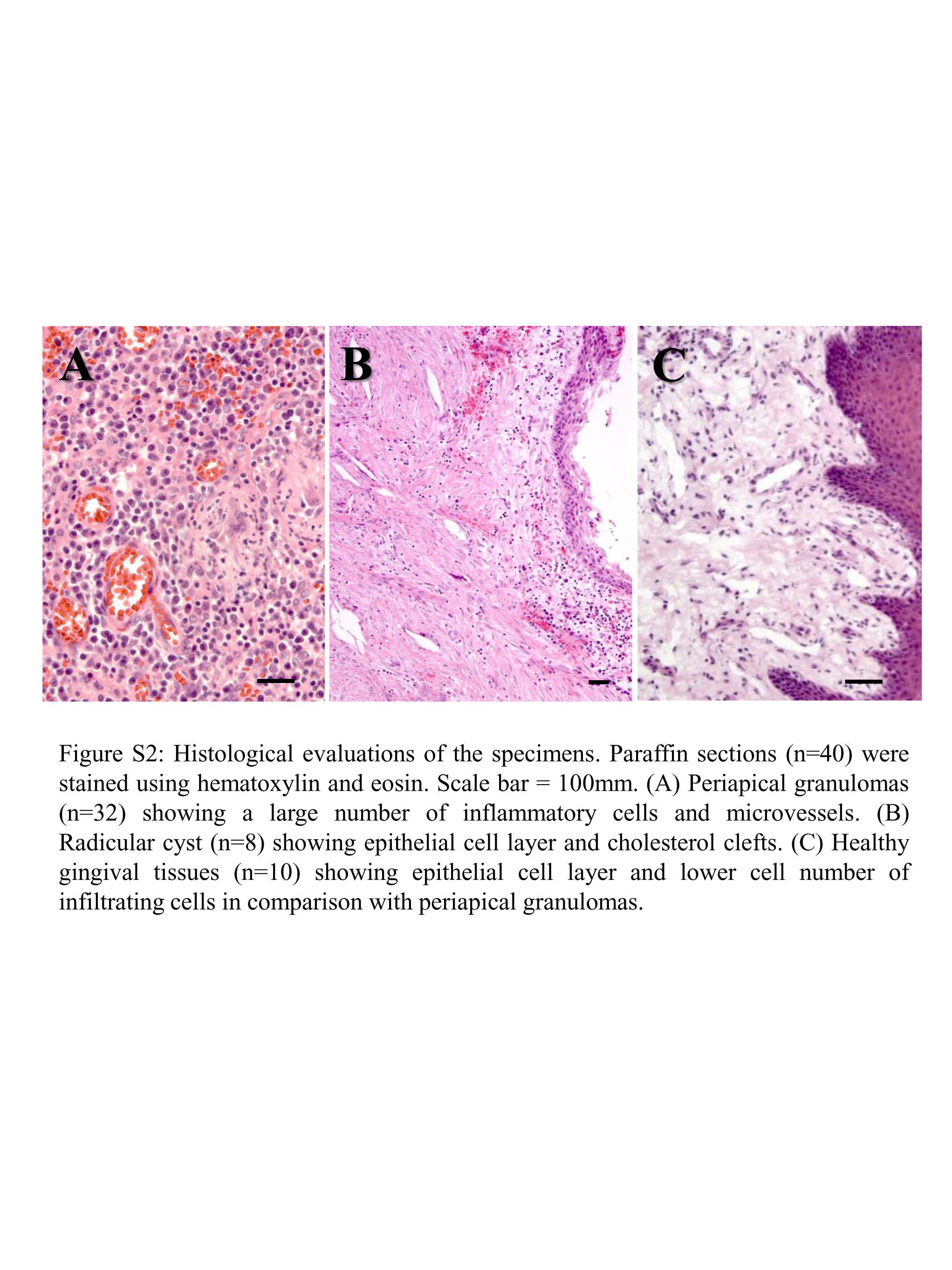

Supplement: S2 Fig — Paraffin sections (n = 40) were stained using hematoxylin and eosin. Scale bar = 100μm. (A) Periapical granulomas (n = 32) showing a large number of inflammatory cells and microvessels. (B) Radicular cyst (n = 8) showing epithelial cell layer and cholesterol clefts. (C) Healthy gingival tissues (n = 10) showing epithelial cell layer and lower cell number of infiltrating cells in comparison with periapical granulomas. (TIF) [file pone.0121548.s002.tif]

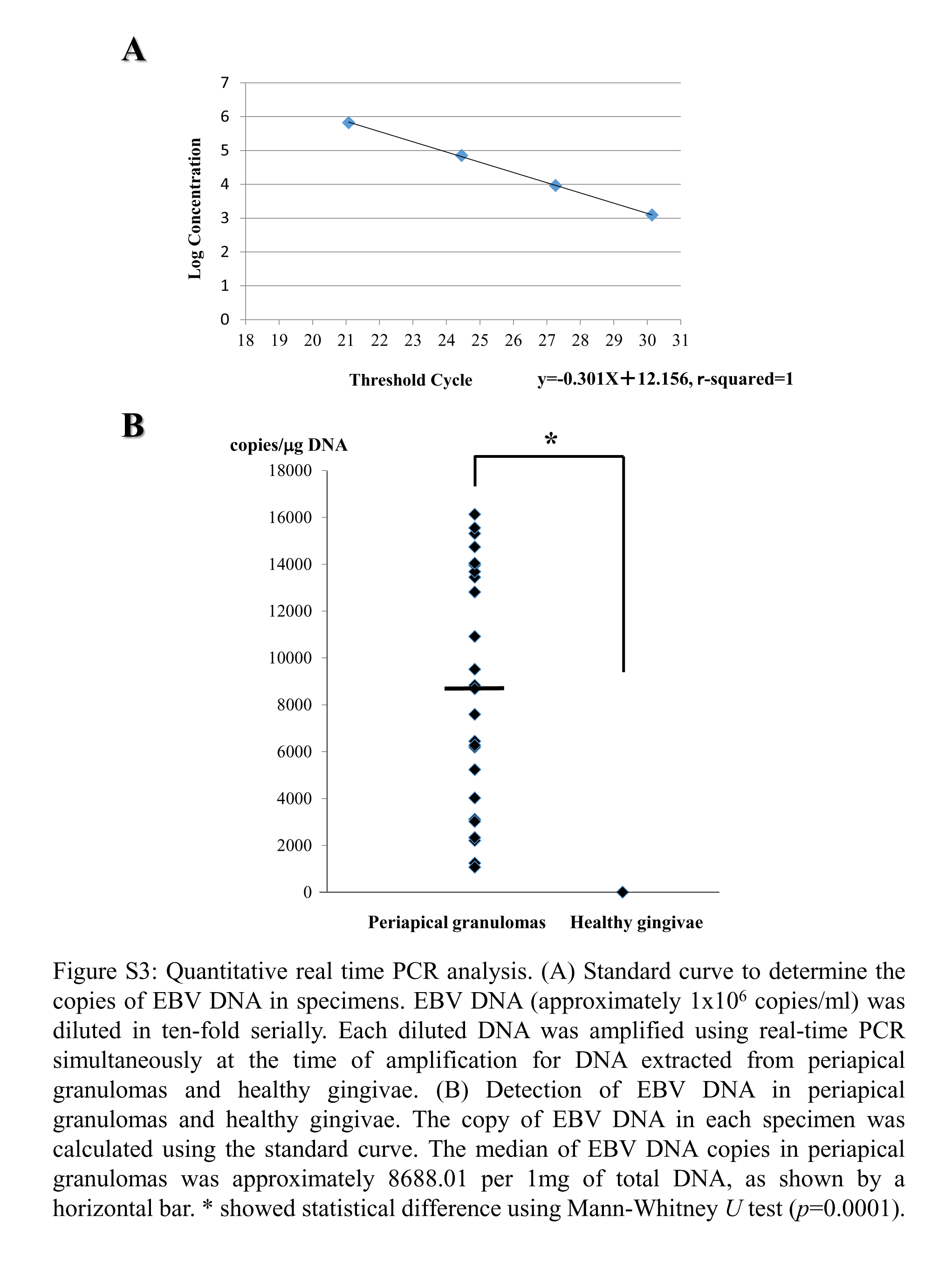

Supplement: S3 Fig — (A) Standard curve to determine the copies of EBV DNA in specimens. EBV DNA (approximately 1x10^6^ copies/μl) was diluted in ten-fold serially. Each diluted DNA was amplified using real-time PCR simultaneously at the time of amplification for DNA extracted from periapical granulomas and healthy gingivae. (B) Detection of EBV DNA in periapical granulomas and healthy gingivae. The copy of EBV DNA in each specimen was calculated using the standard curve. The median of EBV DNA copies in periapical granulomas was approximately 8688.01 per 1μg of total DNA, as shown by a horizontal bar. * showed statistical difference using Mann-Whitney U test (p = 0.0001). (TIF) [file pone.0121548.s003.tif]

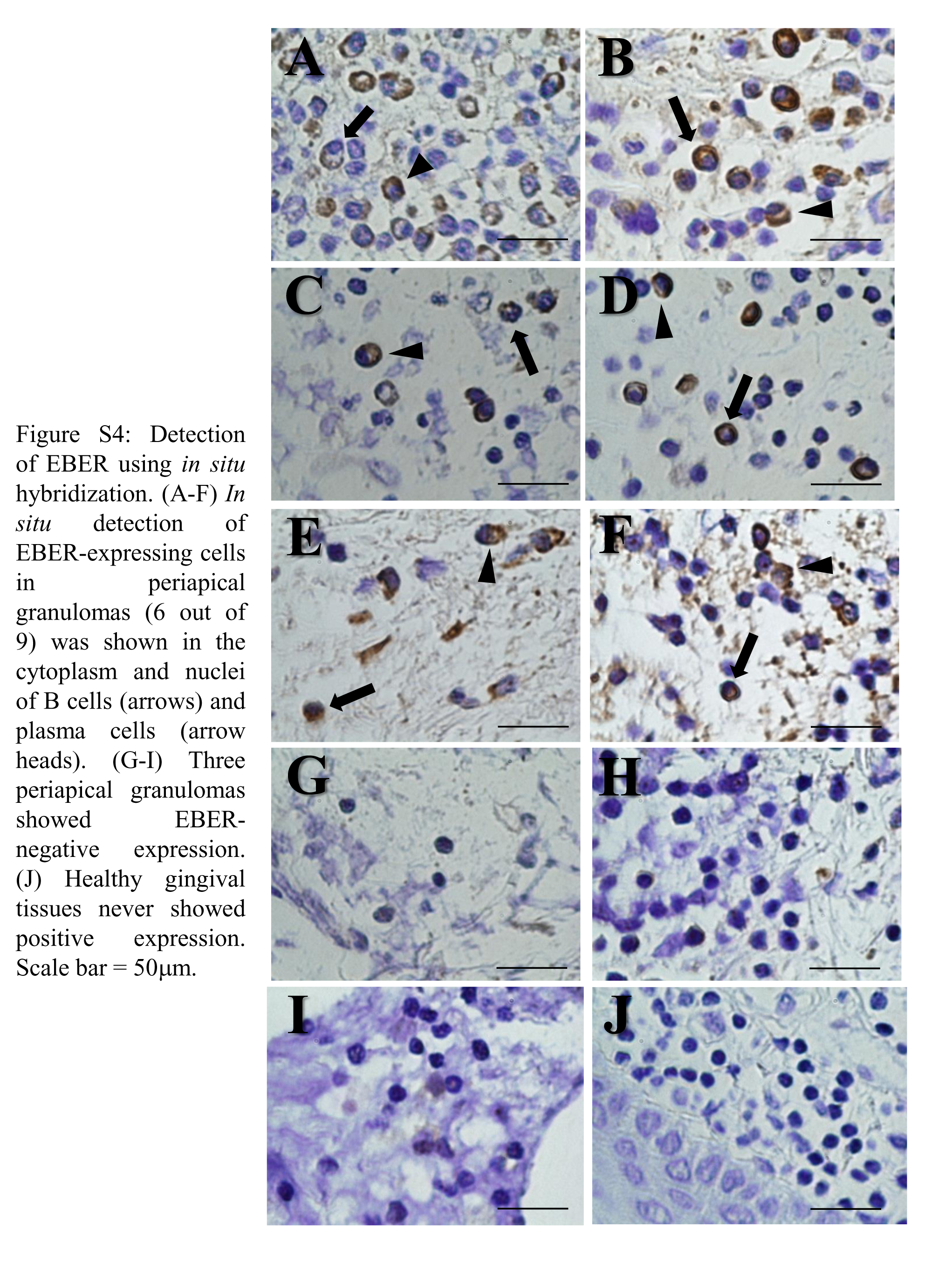

Supplement: S4 Fig — (A-F) In situ detection of EBER-expressing cells in periapical granulomas (6 out of 9) was shown in the cytoplasm and nuclei of B cells (arrows) and plasma cells (arrow heads). (G-I) Three periapical granulomas showed EBER-negative expression. (J) Healthy gingival tissues never showed positive expression. Scale bar = 50μm. (TIF) [file pone.0121548.s004.tif]

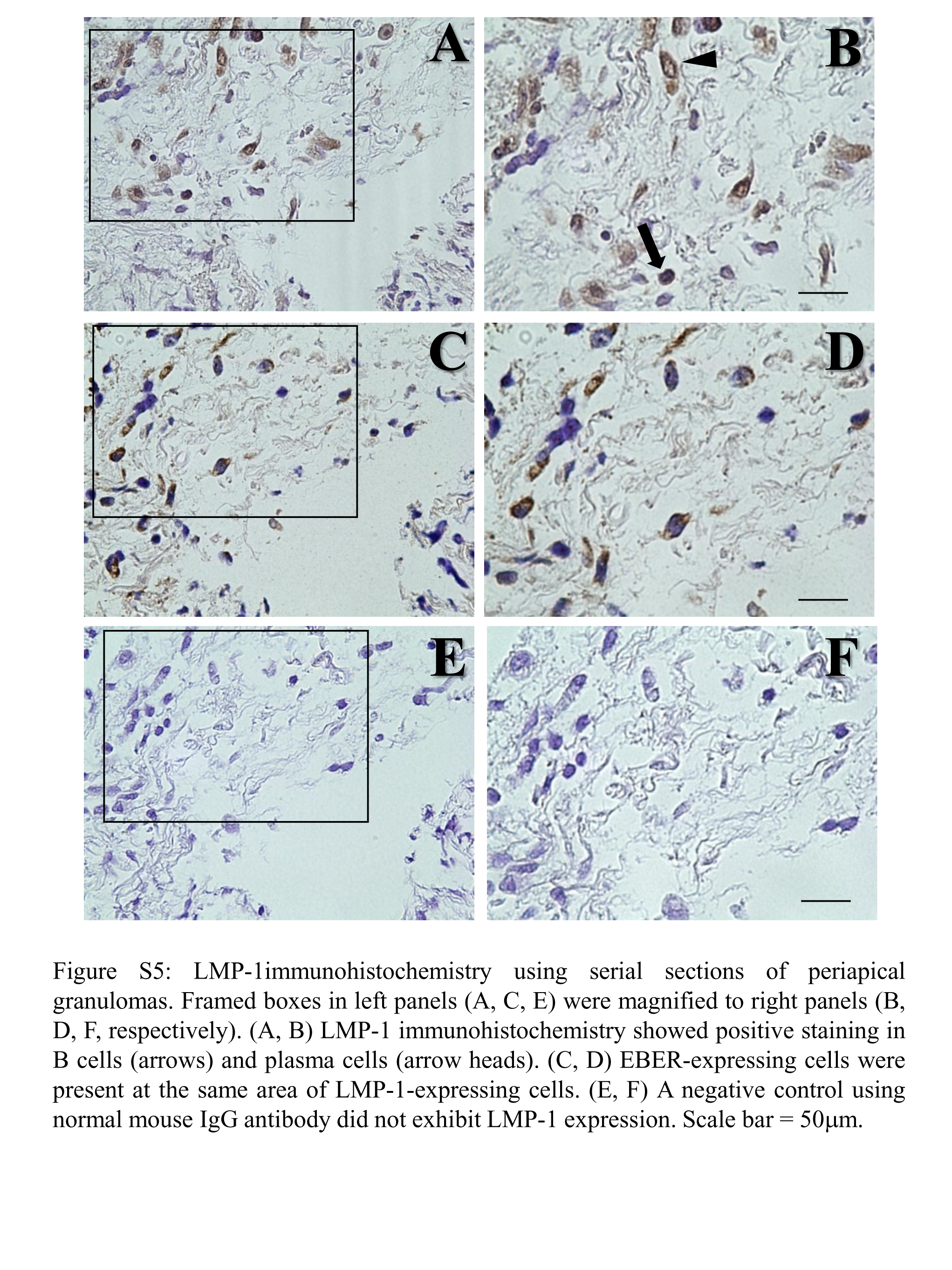

Supplement: S5 Fig — Framed boxes in left panels (A, C, E) were magnified to right panels (B, D, F, respectively). (A, B) LMP-1 immunohistochemistry showed positive staining in B cells (arrows) and plasma cells (arrow heads). (C, D) EBER-expressing cells were present at the same area of LMP-1-expressing cells. (E, F) A negative control using normal mouse IgG antibody did not exhibit LMP-1 expression. Scale bar = 50μm. (TIF) [file pone.0121548.s005.tif]

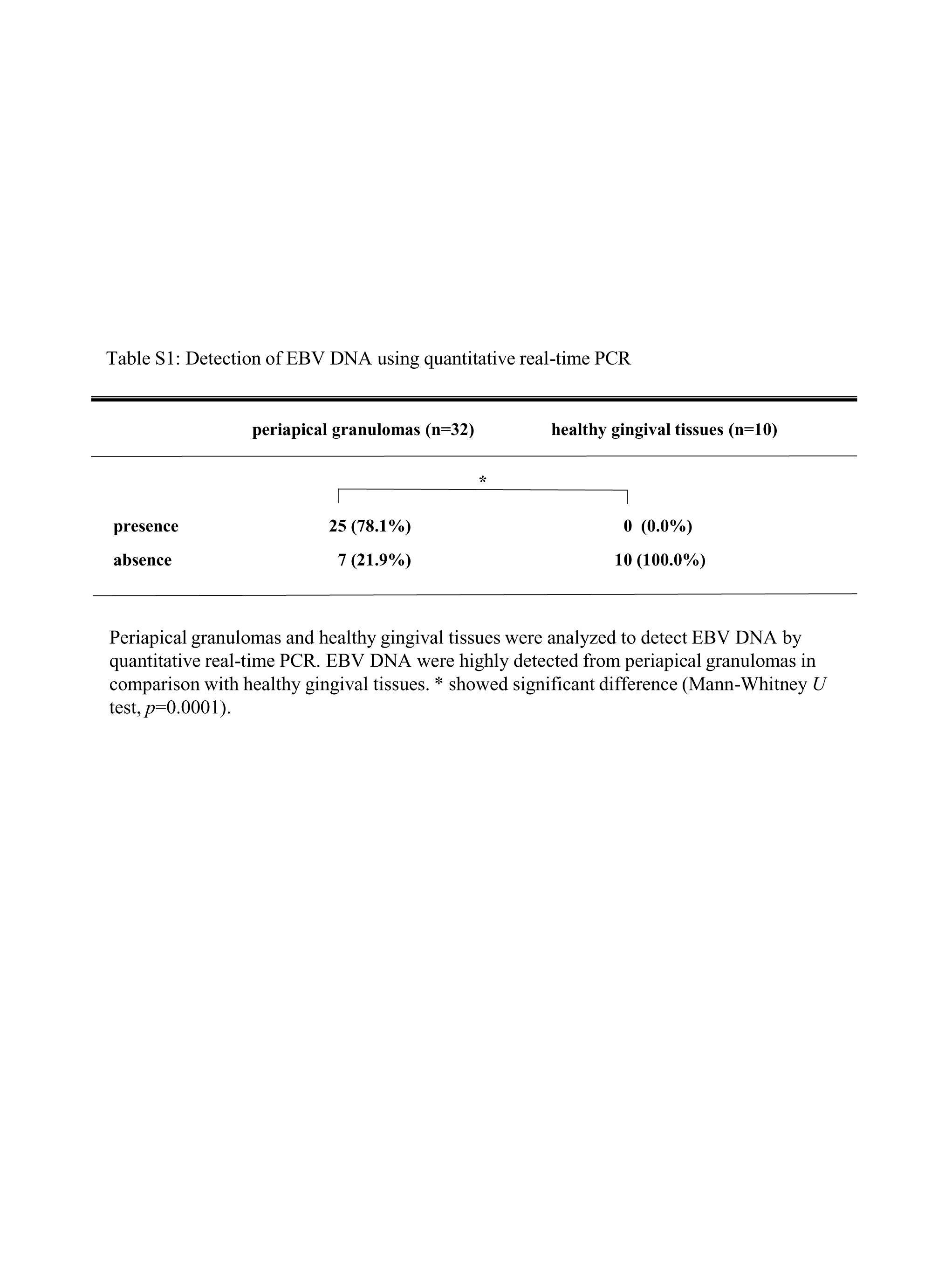

Supplement: S1 Table — Periapical granulomas and healthy gingival tissues were analyzed to detect EBV DNA by quantitative real-time PCR. EBV DNA were highly detected from periapical granulomas in comparison with healthy gingival tissues. * Mann-Whitney U test. (TIF) [file pone.0121548.s006.tif]
